# Cross-reactive immunity against the SARS-CoV-2 Omicron variant is low in pediatric patients with prior COVID-19 or MIS-C

**DOI:** 10.1038/s41467-022-30649-1

**Published:** 2022-05-27

**Authors:** Juanjie Tang, Tanya Novak, Julian Hecker, Gabrielle Grubbs, Fatema Tuz Zahra, Lorenza Bellusci, Sara Pourhashemi, Janet Chou, Kristin Moffitt, Natasha B. Halasa, Stephanie P. Schwartz, Tracie C. Walker, Keiko M. Tarquinio, Matt S. Zinter, Mary A. Staat, Shira J. Gertz, Natalie Z. Cvijanovich, Jennifer E. Schuster, Laura L. Loftis, Bria M. Coates, Elizabeth H. Mack, Katherine Irby, Julie C. Fitzgerald, Courtney M. Rowan, Michele Kong, Heidi R. Flori, Aline B. Maddux, Steven L. Shein, Hillary Crandall, Janet R. Hume, Charlotte V. Hobbs, Adriana H. Tremoulet, Chisato Shimizu, Jane C. Burns, Sabrina R. Chen, Hye Kyung Moon, Christoph Lange, Adrienne G. Randolph, Surender Khurana

**Affiliations:** 1 Division of Viral Products, Center for Biologics Evaluation and Research (CBER), FDA, Silver Spring, MD 20993 USA; 2 Department of Anesthesiology, Critical Care and Pain Medicine, Boston Children’s Hospital, Boston, MA 02115 USA; 3 Department of Anesthesia, Harvard Medical School, Boston, MA 02115 USA; 4 Channing Division of Network Medicine, Department of Medicine, Brigham and Women’s Hospital and Harvard Medical School, Boston, MA 02115 USA; 5 Department of Pediatrics, Boston Children’s Hospital and Harvard Medical School, Boston, MA 02115 USA; 6 Division of Immunology, Boston Children’s Hospital, Harvard Medical School, Boston, MA 02115 USA; 7 Division of Infectious Diseases, Boston Children’s Hospital, Boston, MA 02115 USA; 8 Division of Pediatric Infectious Diseases, Department of Pediatrics, Vanderbilt University Medical Center, Nashville, TN 37232 USA; 9 Department of Pediatrics, University of North Carolina at Chapel Hill Children’s Hospital, Chapel Hill, NC 27514 USA; 10 Division of Critical Care Medicine, Department of Pediatrics, Emory University School of Medicine, Children’s Healthcare of Atlanta, Atlanta, GA 30322 USA; 11 Department of Pediatrics, Divisions of Critical Care and Bone Marrow Transplantation, University of California, San Francisco, San Francisco, CA 94158 USA; 12 Department of Pediatrics, University of Cincinnati, Division of Infectious Diseases, Cincinnati Children’s Hospital Medical Center, Cincinnati, OH 45229 USA; 13 Division of Pediatric Critical Care, Department of Pediatrics, Cooperman Barnabas Medical Center, Livingston, NJ 07039 USA; 14 Division of Critical Care Medicine, UCSF Benioff Children’s Hospital Oakland, Oakland, CA 94609 USA; 15 Division of Pediatric Infectious Diseases, Department of Pediatrics, Children’s Mercy Kansas City, Kansas City, MO 64108 USA; 16 Division of Critical Care Medicine, Department of Pediatrics, Baylor College of Medicine, Houston, TX 77030 USA; 17 Division of Critical Care Medicine, Department of Pediatrics, Northwestern University Feinberg School of Medicine, Ann & Robert H. Lurie Children’s Hospital of Chicago, Chicago, IL 60611 USA; 18 Division of Pediatric Critical Care Medicine, Medical University of South Carolina, Charleston, SC 29425 USA; 19 Section of Pediatric Critical Care, Department of Pediatrics, Arkansas Children’s Hospital, Little Rock, AR 72202 USA; 20 Division of Critical Care, Department of Anesthesiology and Critical Care, The University of Pennsylvania Perelman School of Medicine, Philadelphia, PA 19104 USA; 21 Division of Pediatric Critical Care Medicine, Department of Pediatrics, Indiana University School of Medicine, Riley Hospital for Children, Indianapolis, IN 46202 USA; 22 Division of Pediatric Critical Care Medicine, Department of Pediatrics, University of Alabama at Birmingham, Birmingham, AL 35233 USA; 23 Division of Pediatric Critical Care Medicine, Department of Pediatrics, Mott Children’s Hospital and University of Michigan, Ann Arbor, MI 48109 USA; 24 Department of Pediatrics, Section of Critical Care Medicine, University of Colorado School of Medicine and Children’s Hospital Colorado, Aurora, CO 80045 USA; 25 Division of Pediatric Critical Care Medicine, Rainbow Babies and Children’s Hospital, Cleveland, OH 44106 USA; 26 Division of Pediatric Critical Care, Department of Pediatrics, University of Utah, Salt Lake City, UT 84113 USA; 27 Division of Pediatric Critical Care, University of Minnesota Masonic Children’s Hospital, Minneapolis, MN 55454 USA; 28 Department of Pediatrics, Department of Microbiology, Division of Infectious Diseases, University of Mississippi Medical Center, Jackson, MS 39202 USA; 29 Kawasaki Disease Research Center, Rady Children’s Hospital and Department of Pediatrics, UCSD School of Medicine, La Jolla, CA 92093 USA; 30 Department of Biostatistics, Harvard T.H. Chan School of Public Health, Boston, MA 02115 USA

**Keywords:** Viral infection, SARS-CoV-2, Antibodies, Vaccines

## Abstract

Neutralization capacity of antibodies against Omicron after a prior SARS-CoV-2 infection in children and adolescents is not well studied. Therefore, we evaluated virus-neutralizing capacity against SARS-CoV-2 Alpha, Beta, Gamma, Delta and Omicron variants by age-stratified analyses (<5, 5–11, 12–21 years) in 177 pediatric patients hospitalized with severe acute COVID-19, acute MIS-C, and in convalescent samples of outpatients with mild COVID-19 during 2020 and early 2021. Across all patients, less than 10% show neutralizing antibody titers against Omicron. Children <5 years of age hospitalized with severe acute COVID-19 have lower neutralizing antibodies to SARS-CoV-2 variants compared with patients >5 years of age. As expected, convalescent pediatric COVID-19 and MIS-C cohorts demonstrate higher neutralization titers than hospitalized acute COVID-19 patients. Overall, children and adolescents show some loss of cross-neutralization against all variants, with the most pronounced loss against Omicron. In contrast to SARS-CoV-2 infection, children vaccinated twice demonstrated higher titers against Alpha, Beta, Gamma, Delta and Omicron. These findings can influence transmission, re-infection and the clinical disease outcome from emerging SARS-CoV-2 variants and supports the need for vaccination in children.

## Abstract (web summary)

The antibody response to the SARS-CoV-2 Omicron variant is not well studied in children. Here, the authors provide an age-stratified analysis of SARS-CoV-2 neutralizing capacity of sera from children with acute or convalescent COVID-19 as well as children with multisystem inflammatory syndrome.

## Introduction

Severe acute respiratory syndrome coronavirus 2 (SARS-CoV-2) infection in children and adolescents is usually asymptomatic or causes mild disease, however, they can develop severe manifestations of coronavirus disease 2019 (COVID-19) and are at risk for developing a post-infectious complication called multisystem inflammatory syndrome in children (MIS-C). As of February 2022, the World Health Organization had defined five SARS-CoV-2 variants of concern (VOCs) named Alpha, Beta, Gamma, Delta, and Omicron. The SARS-CoV-2 Omicron variant contains >30 mutations in the SARS-CoV-2 spike protein, allowing rapid spread around the globe, and resulting in large outbreaks in children and adolescents^1–6^. Studies in adults show the SARS-CoV-2 Omicron variant is resistant to neutralizing antibodies after a prior SARS-CoV-2 infection or current SARS-CoV-2 vaccines^2,7–9^.

As of February 2022, children below 5 years are ineligible to receive SARS-CoV-2 vaccination, while those in the age group of 5–11 are eligible to receive 2 vaccine doses and adolescents 12 years and older can get a 3^rd^ vaccine dose in the US. Children are highly impacted by the Omicron outbreak. Despite availability of vaccine for children 5 years and over, vaccination rates remain low especially in patients that developed multisystem inflammatory syndrome in children (MIS-C) related to SARS-CoV-2^10^. Therefore, most children remain susceptible to SARS-CoV-2 infection by emerging SARS-COV-2 variants especially with the highly transmissible Omicron variant^11^, and can potentially transmit to other children and vulnerable populations^12^. Limited knowledge exists regarding SARS-CoV-2 antibody responses in children. Recent studies evaluated immune response following SARS-CoV-2 infection in convalescent children^13^ or asymptomatic group^14^, and did not age stratify children and did not discover age-related differences in different disease cohorts, including acute, severe hospitalized COVID-19 and MIS-C. The antibody response in adults demonstrates diminished ability to neutralize Omicron and other VOCs, but the antibody response in age-stratified children with different diseases categories to VOCs is unclear^8,9,15,16^.

In this study, we evaluated neutralization capacity of serum/plasma samples from three independent pediatric disease cohorts against the SARS-CoV-2 at the time of sample collection and five VOCs: Alpha (B.1.1.7), Gamma (P.1), Beta (B.1.351), Delta (B.1.617.2), and Omicron (B.1.1.529), that were not widely circulating in U.S. The three independent cohorts included children and adolescents with a range of disease severity including patients hospitalized with acute COVID-19 or MIS-C, and convalescent samples from pediatric outpatients who initially had mild COVID-19. To assess the influence of age on the immune response, pediatric cohorts were stratified into <5 years, 5–11 years, and 12–21 years, based on current age stratifications for SARS-CoV-2 vaccines in the U.S.

## Results

**Fig. 1:**
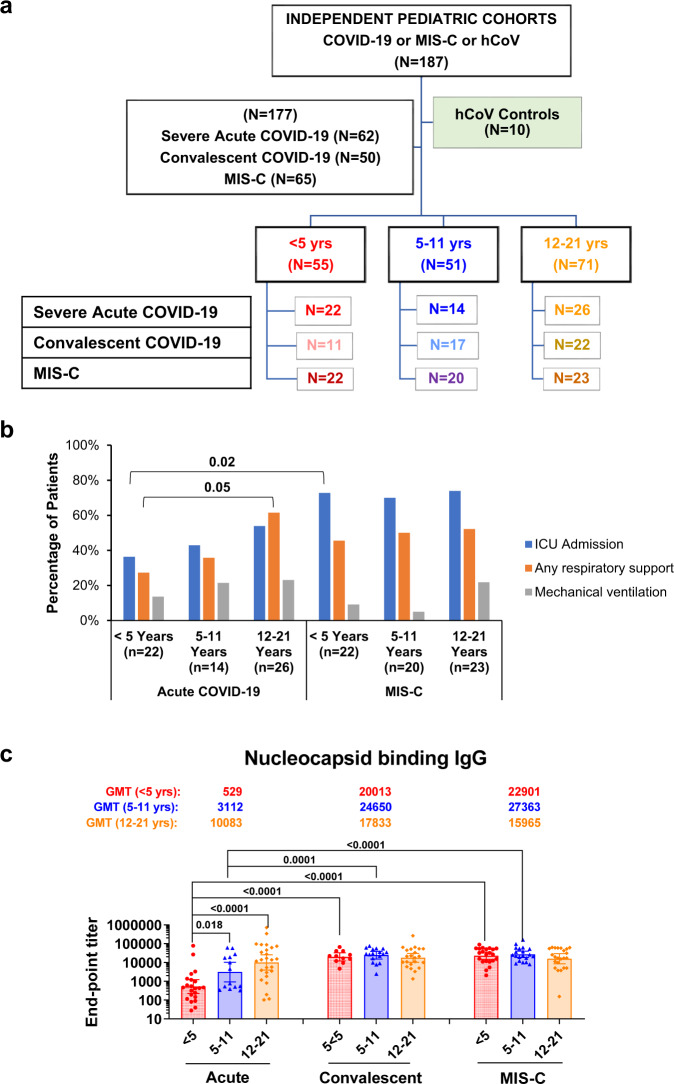
Study design and demographics of children with acute COVID-19 or convalescent COVID-19 or MIS-C and adults with acute COVID-19 and controls. **a** Overview of pediatric cohort, including control (seasonal human coronavirus positive but SARS-CoV-2 negative collected before 2019) and children with acute COVID-19 or convalescent COVID-19 or MIS-C. **b** Percent distribution of hospitalized acute COVID-19 and MIS-C patients admitted to the intensive care unit (ICU), requiring any respiratory support, and receiving mechanical ventilation. **c** SARS-CoV-2 WA1/2020 nucleocapsid binding antibodies in SARS-CoV-2 infected children from either acute COVID-1, convalescent COVID-19 or MIS-C for toddlers (<5 years; in red), pediatric (5–11 years; in blue), or adolescents (12–21 years; in orange). Each serum sample was evaluated in IgG-ELISA in duplicate to determine the nucleocapsid-binding IgG end-point titer. The height of bars and numbers over the bars indicate the IgG GMTs, and the whiskers indicate 95% confidence intervals. Statistical differences between age groups within each disease category or between different disease category within each age group were analyzed by R and the two-sided statistically significant p-values are shown. The *p*-values are not corrected for multiple comparisons. Source data are provided as a Source Data file.

Antibody profiling was performed on the samples from 177 children hospitalized with either acute COVID-19 or MIS-C, or outpatient mild convalescent COVID-19 (Fig. 1a and Supplementary Tables S1 and S2). Children <5 years old hospitalized with acute COVID-19 had significantly less ICU admissions compared to MIS-C patients (*p* = 0.02) (Fig. 1b). Acute children <5 years old also required significantly less respiratory support than older children (12–21) with the same acute illness (*p* = 0.05). There were no differences in disease severity based on ICU admission, respiratory support, or mechanical ventilation among different MIS-C age groups. None of these children were vaccinated.

SARS-CoV-2 WA1 nucleocapsid binding IgG were observed in all the 177 SARS-CoV-2 infected children (Fig. 1c). In children hospitalized with acute COVID-19, an age-stratified nucleocapsid binding IgG titer was observed, with younger children (<5 years old) showing lower nucleocapsid IgG compared with older children (≥5 years). No age difference was observed for nucleocapsid IgG in children with either outpatient mild convalescent COVID-19 or with MIS-C. Younger children (<5 years) with convalescent COVID-19 or MIS-C demonstrated higher nucleocapsid IgG titers compared with <5 years children with acute COVID-19.

Pseudovirion neutralization assay (PsVNA) was used to measure the antibody neutralization activity of the children’s samples against the predominant SARS-CoV-2 WA1 strain and the five VOCs (Supplementary Table S3). SARS-CoV-2 neutralizing activity measured by this qualified PsVNA correlated with PRNT (plaque reduction neutralization test with authentic SARS-CoV-2 virus) in previous studies^17,18^. As a control, samples from 10 critically ill children positive for seasonal coronaviruses collected prior to 2019 did not demonstrate neutralization titers against SARS-CoV-2 (Source Data file).

**Fig. 2:**
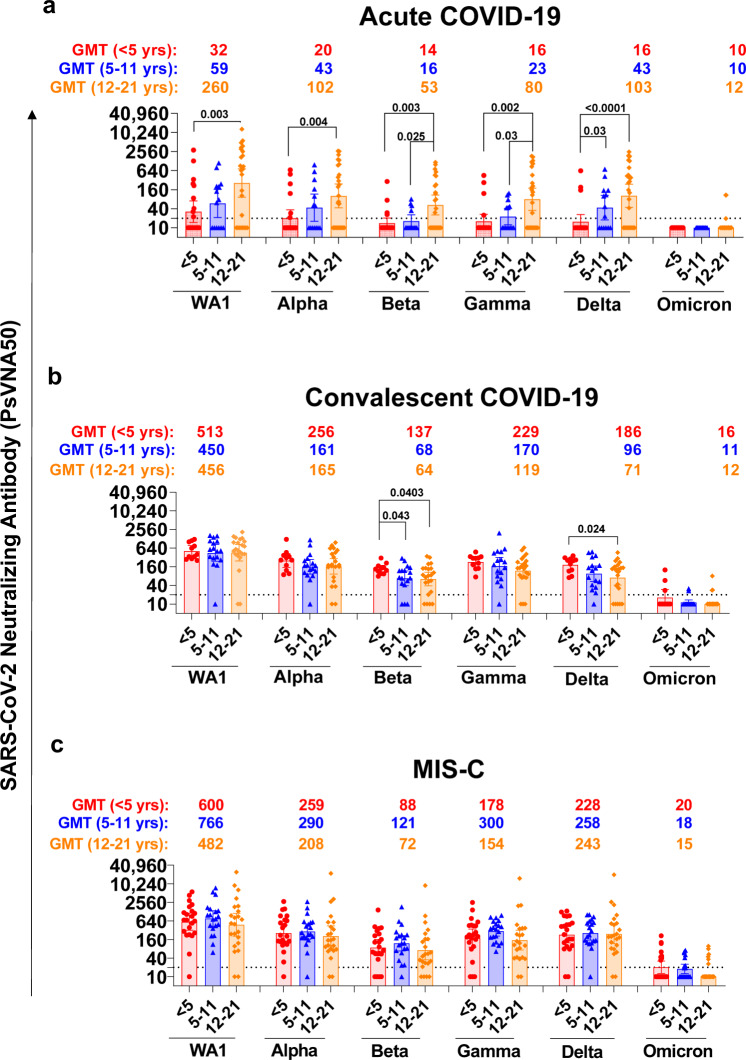
Neutralizing antibody titers of serum/plasma from children with COVID-19 or MIS-C against SARS-CoV-2 WA1 and VOCs. **a**–**c** Geometric mean titer (GMT) values ± 95% confidence interval for pseudovirus neutralization assay (PsVNA) neutralization titers (PsVNA50; 50% virus neutralization titers) for serum/plasma from 177 children with either acute COVID-19 (**a**), convalescent COVID-19 (**b**) or inpatients with MIS-C (**c**), against ancestral SARS-CoV-2 WA1 and VOCs: Alpha (B.1.1.7), Beta (B.1.351), Gamma (P.1), Delta (B.1.617.2), and Omicron (B.1.1.529) for toddlers (<5 years; in red), pediatric (5–11 years; in blue), adolescents (12–21 years; in orange) with COVID-19 or MIS-C as determined by PsVNA in 293-ACE2-TMPRSS2 cells. GMT values for PsVNA50 titers are color coded for each of the age group. The horizontal dashed line indicates the limit of detection for the neutralization assay (PsVNA50 of 1:20). The samples that did not neutralize SARS-CoV-2 at 1:20 serum dilution was given a PsVNA50 value of 10 for graphic representation and statistical analysis. The PsVNA is a qualified assay where all samples are run with a set of internal standards in every plate of the neutralization assay and conforms with assay performance. All PsVNA experiments were performed in duplicate and the researchers performing the assay were blinded to sample identity. The variations for duplicate runs were <7%. The data shown are average values of two experimental runs. Statistical differences were analyzed in R (version 4.1.2) using a permutation-based approach and the two-sided statistically significant *p*-values are shown. The *p*-values are not corrected for multiple comparisons. Source data are provided as a Source Data file.

The 177 post-SARS-CoV-2 infection samples demonstrated variable PsVNA50 titers (ranged from 1:10 to 1:24,430 for a sample dilution that resulted in 50% virus neutralization) against ancestral WA1 strain (Fig. 2 and Supplementary Fig. S1). A steady increase of neutralizing antibodies was observed as age increased from young children (<5 years) with geometric mean titers (GMT) of 1:32 to 1:260 for adolescents (12–21 years) against the ancestral WA1 strain (Fig. 2a) in children with acute COVID-19. Adolescents (aged 12–21) with acute COVID-19 showed 4.4 to 8.1-fold higher WA1 neutralization GMT compared with patients ≤11 years of age. However, samples from inpatients hospitalized with MIS-C or convalescent samples from non-hospitalized outpatient children that recovered from mild COVID-19 (Convalescent COVID-19), demonstrated an increase in WA1 neutralizing antibodies in younger children such that PsVNA50 titers were similar across all age groups (Fig. 2b, c, and Supplementary Figs. S1a–c and S2b, c). In younger patients (≤11 years old), as expected, those with MIS-C (GMT from 1:600 to 1:766) or convalescent COVID-19 (GMT from 1:450 to 1:513) demonstrated 13 to 19-fold higher PsVNA50 GMT against WA1 than children with acute COVID-19 (GMT from 1:32 to 1:59) (Fig. 2 and Supplementary Fig. S2a, b).

For the acute COVID-19 patients, neutralization titers against VOCs demonstrated a similar age-stratified trend from low PsVNA50 titers in younger children (<5 years) to higher titers in adolescents (Fig. 2a). In contrast to PsVNA50 titers against WA1, neutralization of the five VOCs was significantly reduced across all age groups (Supplementary Fig. S1a). The highest reduction in PsVNA50 titers was observed against Omicron such that none (0/36) of the younger children (≤11 years; GMT of 1:10) and only 2/26 adolescents (12–21 years; GMT of 1:12) demonstrated PsVNA50 titers above the seropositive cut-off of PsVNA50 titer of 1:20 against Omicron (Fig. 2a and Supplementary Fig. S1a).

For convalescent COVID-19 patients, although neutralization titers against WA1 among the age groups were comparable (GMT of 1:450 to 1:513), an age-dependent trend of decreasing neutralization titers was observed against Delta and Beta VOCs, with ~2-fold higher GMT in younger children (<5 years) compared with adolescents (Fig. 2b). Similar to WA1, neutralization titers of VOCs were significantly higher in convalescent individuals than acute-COVID-19 patients for younger children (≤11 years) but not in adolescents (12–21 years) (Supplementary Fig. S2). The greatest reduction in PsVNA50 titers compared with WA1 was observed against Omicron (32–41-fold), followed by Beta (4–7-fold) and then Delta (3–6-fold) across all convalescent COVID-19 age groups (Supplementary Fig. S1b). Most importantly, only 16% (8/50) convalescent children across age groups demonstrated seropositivity (PsVNA50 > 1:20) against Omicron (PsVNA50 ranging from 1:10 to 1:127) (Fig. 2b).

For MIS-C patients, there was a consistent drop in neutralization titers against VOC with similar patterns across all ages (Fig. 2c and Supplementary Fig. S1c). Moreover, in children ≤11 years old, similar to WA1, the absolute PsVNA50 titers against the five VOC in MIS-C patients demonstrated higher neutralizing titers compared with acute COVID-19 patients, but not in adolescents (Supplementary Fig. S2c). The antibody response of the MIS-C patients and those with convalescent COVID-19 were similar across age groups.

**Fig. 3:**
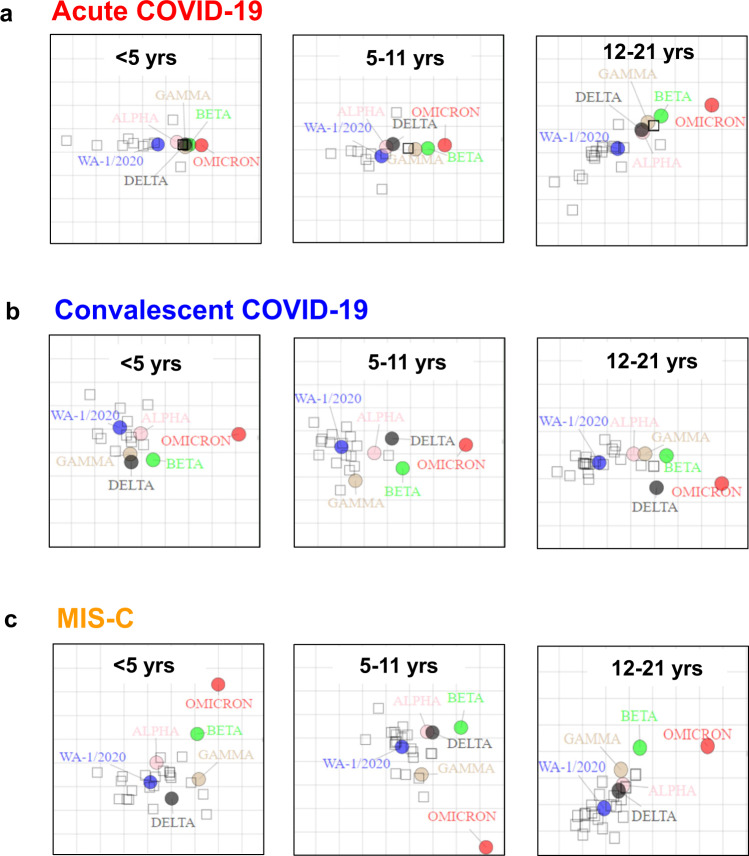
Antigenic cartography of different age group children with acute COVID-19 vs convalescent COVID-19 vs MIS-C against SARS-CoV-2 WA1 and five VOCs. Individual antigenic maps were generated for each disease cohort in young children (<5 years), school-age children (5–11 years), and adolescent (12–21 years), with either acute COVID-19 (**a**), convalescent COVID-19 (**b**) or MIS-C (**c**), against SARS-CoV-2 WA1 (blue) or the VOCs Alpha (light orange circle), Beta (green circle), Gamma (brown circle), Delta (black circle) and Omicron (red circle). Black diamonds correspond to each individual sera/plasma. Each grid square corresponds to 2-fold dilution in the neutralization assay. Source data are provided as a Source Data file.

Antigenic cartography was performed to explore how the different age and disease categories in these pediatric cohorts distinguish neutralization of the SARS-CoV-2 variants (Fig. 3). For acute COVID-19, the variants were tightly clustered closer to the ancestral WA1 strain in younger children (≤11 years), while the distribution was more heterogeneous for acute COVID-19 adolescents, especially with distance between WA1 and Omicron. Across all age groups, the convalescent COVID-19 and MIS-C samples were heterogenous with Alpha, Gamma, and Delta VOCs clustered around WA1, with small distance between WA1 and Beta for convalescent COVID-19 (5 years and above) and all MIS-C patients. The antigenic distances between Omicron and WA1 were large for all convalescent COVID-19 and MIS-C across all age cohorts, in agreement with the neutralization titers observed in Fig. 2.

Although we do not have serial samples on all hospitalized acute COVID-19 patients, it is possible that the younger children hospitalized with COVID-19 had a less robust neutralizing antibody response due to lower disease severity, which likely improved during convalescence (convalescent COVID-19, or MIS-C which presents weeks after initial infection), such that their capacity to neutralize the ancestral SARS-CoV-2 WA1 strain and the Alpha, Beta, Gamma, and Delta VOCs is similar to adolescents with COVID-19 or MIS-C. Importantly, a majority (>90%) of the children evaluated in this study did not demonstrate neutralizing antibodies against the Omicron variant.

**Fig. 4:**
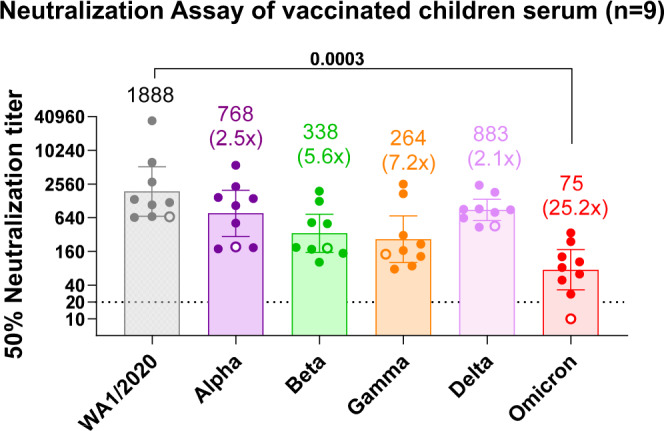
Neutralization by post-vaccination serum from children against SARS-CoV-2 WA1/2020 strain and variants of concern. Serum samples following one (*n* = 2) or two (*n* = 7) doses of SARS-CoV-2 mRNA vaccine were obtained from nine healthy children without any co-morbidities. Neutralization assays were performed with the use of pseudoviruses harboring the SARS-CoV-2 spike proteins of the WA1/2020 vaccine strain or VOCs: Alpha, Beta, Gamma, Delta, or the Omicron variants. The assay of each individual sample (circles) was performed in duplicate to determine the 50% neutralization titer against the indicated pseudovirus. One child who got only a single vaccination is shown as an open symbol. The heights of the bars and the numbers over the bars indicate the geometric mean titers, and the whiskers indicate 95% confidence intervals. The numbers in parentheses indicate the average decrease in neutralization titer of the indicated variants as compared with that of the WA1/2020 virus. The horizontal dashed line indicates the limit of detection for the neutralization assay (PsVNA50 of 20). Differences between SARS-CoV-2 strains were analyzed by R and the two-sided statistically significant p-values are shown. The *p*-values are not corrected for multiple comparisons. Source data are provided as a Source Data file.

To determine if vaccination of naïve (SARS-CoV-2 negative) adolescents leads to greater cross-neutralization of VOCs including Omicron, we evaluated neutralizing antibody response in a comparator group of children (median age of 16.9 years; range 15.9–19.0 years) who were vaccinated with two doses of either Pfizer (BNT16b2) or Moderna mRNA vaccine (*n* = 7) or a single dose of either Pfizer or Moderna (*n* = 2). Serum samples were obtained between 19 and 163 days (median of 50 days) after vaccination. In contrast to SARS-CoV-2 infection, children vaccinated once or twice demonstrated higher titers against vaccine-homologous WA1 strain (GMT 1:1888) as well all five VOCs, Alpha, Beta, Gamma, Delta, and Omicron (Fig. 4). Largest reduction (25.2-fold) of neutralization titers in vaccinated children was observed against the Omicron variant, however, importantly, all 8 of the 9 children who received two doses of mRNA vaccine still showed PsVNA50 titers >1:20 against Omicron.

## Discussion

Understanding the pediatric antibody response against SARS-CoV-2 Omicron and emerging variants is critical for disease management, development of therapeutics, and refinement of vaccines in this under-studied population. This study of children and adolescents ≤21 years old that were previously infected with SARS-CoV-2 between April 2020 and March 2021 revealed an age-dependent effect in the neutralization of five different SARS-CoV-2 VOCs. The most prominently reduced antibody neutralization occurred in the youngest children with acute COVID-19 compared with older age groups. MIS-C patients and convalescent outpatients who had mild COVID-19 showed similar responses across all ages. Overall, results suggest a differentially evolving quantitative and qualitative neutralizing response to SARS-CoV-2 and VOCs in children less than 5 years old and who are currently ineligible for vaccination. This is especially important as children in this study were infected with SARS-CoV-2 prior to the circulation of Delta or Omicron did not have neutralizing antibodies against Omicron and therefore are likely susceptible to re-infection with the Omicron variant.

During acute COVID-19, an age-dependent increase in neutralizing antibodies against SARS-CoV-2 WA1 and VOC was observed from young children to adolescents. Recently, cellular immune profiling demonstrated that systemic immune response in the blood of children is characterized by a more naive state compared with a more memory-based immune repertoire in adults^19^. Since younger children (<5 years) have lower percentages of memory B cells than adolescents, they generate a primary immune response after SARS-CoV-2 infection, which develops slower than a recall response, as was observed for lower antibodies during acute COVID-19 in younger children (<5 years). The timeframe of symptom onset was also considered in understanding the acute immune response. In our acute COVID-19 cohort, the median timeframe between symptom onset and their sample being collected was 3 days (IQR 2, 6 days) for children <5 years old, 3.5 days (IQR 2, 5 days) for children 5–11 years old, and 6 days (IQR 2, 10 days) for adolescents giving the older children only a slightly longer timeframe to start producing antibodies, although there was no significant difference (*p* value of 0.2–1.0) between different age cohorts.

During post-infectious MIS-C or convalescence COVID-19, children of all ages demonstrated similar neutralization capacity to the WA1 strain, however, the GMT against the Beta and Delta VOC were higher in younger children (<5 years) compared with convalescent COVID-19 adolescents (12–21 years). One possible explanation for these qualitative antibody differences against VOCs during convalescent COVID-19 between age groups could be due to the original antigenic sin (OAS) hypothesis, whereby older children have B-cell memory due to prior exposure to seasonal coronaviruses, especially in SARS-CoV-2 spike S2 domain as observed in older children, adults, and elderly^20–23^. Recently, we observed anti-S2 cross-reactivity in naive older children but not in the younger children (<4 years old), who share homology with HKU1, 229E, and OC43^23,24^. OAS was also observed in mice immunization studies with seasonal CoV followed by SARS-CoV-2 spike^25^. Most of these cross-reactive antibodies do not neutralize SARS-CoV-2, as was observed with samples from 10 children with seasonal CoVs in our study. It is expected that cross-reactive memory B cells will be recalled shortly after SARS-CoV-2 infection (likely with a WA1 or Alpha-like strain in this cohort) primarily in immune-exposed older children, that results in a more focused recall antibody response, which potentially diminishes the immune system’s ability to recognize and neutralize SARS-CoV-2 variants like Beta and Delta in older children. Since immune system in younger children (<5 years) is more naive than adolescents, they generate a more diverse primary antibody response following SARS-CoV-2 infection, which demonstrates a robust, broad anti-SARS-CoV-2 immunity capable of effectively neutralizing variants like Beta and Delta.

We evaluated the convalescent antibody response in children under 5 years old who presented with mild symptoms of SARS-CoV-2 infection or were asymptomatic and tested PCR positive after a family member was infected. These patients were recruited as a control group for those at risk of MIS-C. Our study showed that these young children without access to COVID-19 vaccination had a robust antibody response against most SARS-CoV-2 variants during convalescence. These samples came from time periods prior to circulation of Delta or Omicron variants in the US and vaccines were not available for adolescents, so patients were highly unlikely to be exposed to these variants and were not administered SARS-CoV-2 vaccine. The MIS-C cohort, which is also a post-infectious convalescent state and rare complication, showed a similar pattern to that of the convalescent patients who did not develop MIS-C. The majority of MIS-C patients were in the ICU requiring respiratory support at the time the sample was taken, but despite this, their antibody response was very similar to that of the convalescent children with mild disease who were never hospitalized. And although IVIG was administered to an average of 59.8% MIS-C patients before samples were collected, their antibody levels were similar to those in convalescent COVID-19 patients and are not thought to impact the results. To support this, our previous studies have shown that IVIG produced by US manufacturers up to March 2021 did not contain anti-SARS-CoV-2 antibodies since they were made from plasma collected prior to 2020^17^.

To our knowledge, this is the first evaluation of antibody response to Omicron in convalescent COVID-19 and MIS-C pediatric patients, who are potentially at risk of re-infection with Omicron or newly emerging SARS-CoV-2 variants, as has been observed in adults^26^. A significant strength of this study is that the pediatric acute and MIS-C samples were from a U.S. national multicenter cohort collected during the pandemic, increasing generalizability. All samples were collected between April 2020 and March 2021. In the US, the WA1 strain had been circulating since January 2020 and as of 11 March 2021, the majority of emerging VOC cases were due to the Alpha strain, followed by 108 cases of Beta, and only 17 cases of Gamma^27^. One study limitation is that we do not have patients’ SARS-CoV-2 viral sequencing to verify or explain the variability of the different pediatric age groups in successfully neutralizing the SARS-CoV-2 variants. Lastly, since the acute COVID-19 and MIS-C patients were hospitalized, we chose to statistically control for disease severity when comparing antibody levels. Another limitation is cohort of vaccinated children is small, as well as skewed towards the older (>15) age group. However, the neutralizing antibody response measured in our study mimics to those observed in younger children against the vaccine-matched strain^28^.

In conclusion, our findings suggest that the antibodies produced by previous SARS-CoV-2 infection in pediatric population do not neutralize the currently circulating Omicron variant and therefore they potentially remain susceptible to re-infection with Omicron. Vaccine induced a much broader neutralizing antibody response against VOCs in naïve children compared with the natural immunity induced following SARS-CoV-2 infection. Our study highlights a decrement in the antibody response to VOCs in children after natural infection that was most striking for the Omicron variant whereas the breadth of the antibody response was more robust in a control group of vaccinated children. Despite availability in the U.S. since October 2021, vaccine uptake in children ages 5–11 years is overall low as of March 2022^10^. Our study highlights the importance of vaccinating children and younger adolescents even with preexisting antibody immunity by an earlier SARS-CoV-2 strain to prevent severe disease in children from Omicron and future infections^28–30^. These findings have direct implications for developing age-targeted strategies for testing, disease mitigation, vaccination, and protecting this vulnerable population.

## Methods

### Study design

The objective of this study was to investigate the neutralizing capacity of serum or plasma from children of age-stratified groups with acute hospitalized COVID-19 or mild outpatient convalescent COVID-19 or hospitalized MIS-C with SARS-CoV-2 VOCs: Alpha (B.1.1.7), Beta (B.1.351), Gamma (P.1), Delta (B.1.617.2), and Omicron (B.1.1.529).

The 177 U.S. pediatric patients (mean age 9 years [IQR 3.6, 14.8 years]) enrolled comprised 3 independent cohorts: 62 hospitalized with acute COVID-19, 65 hospitalized with MIS-C, and 50 non-hospitalized children with mild COVID-19 with convalescent samples (≥30 days post-acute) enrolled as an at-risk for MIS-C control group (Fig. 1). MIS-C and acute COVID-19 were defined using U.S. Centers for Disease Control and Prevention (CDC) case definitions (criteria listed below). Patients were <5 years (*n* = 62: 22 acute COVID-19, 14 convalescent COVID-19, 26 MIS-C), 5–11 years (*n* = 50: 11 acute COVID-19, 17 convalescent COVID-19, 22 MIS-C), and 12–21 years old (*n* = 65: 22 acute COVID-19, 20 convalescent COVID-19, 23 MIS-C). All acute and convalescent COVID-19 pediatric patients had SARS-CoV-2 detected by reverse transcriptase quantitative PCR (RT-qPCR) and MIS-C patients had positive SARS-CoV-2 antibody and/or RT-qPCR tests. Demographic, clinical and laboratory data are summarized in Tables S1 and S2. Samples from the three pediatric cohorts (acute COVID-19 vs convalescent COVID-19 vs MIS-C) were collected from a prospectively enrolling multicenter study (Overcoming COVID-19). Children’s acute COVID-19 samples (serum or plasma) were collected as early as possible during hospitalization and/or study enrollment.

MIS-C is a hyperinflammatory syndrome that occurs ~3–6 weeks post-SARS-CoV-2 infection in children and patients in this cohort were asymptomatic or had mild illness upon initial infection. As a control group for MIS-C, we collected samples from outpatient children and adolescents with a positive PCR test for SARS-CoV-2 and asymptomatic or mild illness (never hospitalized) ~3–6 weeks after their positive test. Because MIS-C is rare (~2–3 per 10,000 SARS-CoV-2 infections in US) and it not possible to identify patients in advance, it is not feasible to get baseline (during initial infection) samples in most MIS-C patients^31^.

Serum samples were also obtained from 9 naïve (SARS-CoV-2 negative) children (16.9 years median age; age range 15.9–19.0 years) who were vaccinated with one or two doses of Pfizer (BNT16b2) (*n* = 7) or Moderna (*n* = 2) mRNA vaccine. Samples were collected between 19 and 163 days (median of 50 days) post-vaccination from these children.

### Clinical study and case definitions

The Overcoming COVID-19 Network studies severe complications of COVID-19 in children and adolescents and their public health surveillance work has been described previously^32^; a subgroup of 20 sites in 18 U.S. states participate in the Overcoming COVID-19 Immunobiology Study. Study sites relied on a single IRB at Boston Children’s Hospital under Protocol Number #IRB-P00033157, and informed consent was obtained from at least one parent or legal guardian. Samples from the Boston Children’s Hospital COVID-19 Biobank Taking on COVID-19 Together Protocol included consented patient samples and de-identified samples obtained with IRB-approved waiver of consent under #IRB-P00035409. Seasonal coronavirus controls samples were from studies prior to the COVID-19 pandemic^33^.

All pediatric patients were <21 years old with confirmed SARS-CoV-2 positive PCR or antibody testing. Patients with immune compromising conditions that could impair antibody responses were excluded, as were patients with life support limitations or end stage lung disease. Hospitalized patients with COVID-19-related complications were hospitalized for acute COVID-19 or MIS-C as defined below and samples were collected acutely as early into their hospital course as possible. Convalescent patients were outpatient SARS-CoV-2 PCR positive patients with mild or no symptoms, presenting to the Emergency Department or clinics affiliated with Boston Children’s Hospital who returned for a blood draw approximately 4-6 weeks later. Data collection included demographic information including race and ethnicity, past medical history including chronic health conditions, SARS-CoV-2 testing results, clinical diagnosis of acute COVID-19 or MIS-C, and hospital course including discharge outcome.

### Clinical cohorts

In this study, 62 pediatric patients were hospitalized with acute COVID-19. This was defined as having signs or symptoms that could be associated with early SARS-CoV-2 infection accompanied by a positive real-time polymerase chain reaction (RT-PCR) test for severe acute respiratory syndrome coronavirus 2 (SARS-CoV-2) as defined by the U.S. Centers for Disease Control and Prevention (CDC) as listed on their website (https://wwwn.cdc.gov/nndss/conditions/coronavirus-disease-2019-covid-19/case-definition/2020/ approved 5 April 2020).

- At least two of the following symptoms: fever (measured or subjective), chills, rigors, myalgia, headache, sore throat, new olfactory, and taste disorder(s); AND
- At least one of the following symptoms: cough, shortness of breath, or difficulty breathing; OR
- Severe respiratory illness with at least one of the following:
  - Clinical or radiographic evidence of pneumonia, OR
  - Acute respiratory distress syndrome (ARDS).
  AND
- No alternative more likely diagnosis

MIS-C patients met the criteria for Multisystem Inflammatory Syndrome in Children as defined by the CDC^32^ as listed on their website (https://www.cdc.gov/mis-c/hcp/ published 5/14/2021) including:

- An individual aged <21 years presenting with fever*, laboratory evidence of inflammation**, and evidence of clinically severe illness requiring hospitalization, with multisystem (≥2) organ involvement (cardiac, renal, respiratory, hematologic, gastrointestinal, dermatologic or neurological); AND
- No alternative plausible diagnoses; AND
- Positive for current or recent SARS-CoV-2 infection by RT-PCR, serology, or antigen test; or exposure to a suspected or confirmed COVID-19 case within the 4 weeks prior to the onset of symptoms.
  *Fever ≥38.0° C for ≥24 h, or report of subjective fever lasting ≥24 h **Including, but not limited to, one or more of the following: an elevated C-reactive protein (CRP), erythrocyte sedimentation rate (ESR), fibrinogen, procalcitonin, d-dimer, ferritin, lactic acid dehydrogenase (LDH), or interleukin 6 (IL-6), elevated neutrophils, reduced lymphocytes, and low albumin

Convalescent COVID-19 samples were collected from outpatients presenting to the emergency department or clinics between approximately 1–3 months after initial positive SARS-CoV-2 RT-PCR who had asymptomatic SARS-CoV-2 infection or mild acute COVID-19 infection (received no oxygen or other support) at the time of the positive PCR. Patients that later met criteria for MIS-C or developed acute COVID-19 were excluded from this cohort. Patients were never hospitalized for SARS-CoV-2-related complications prior to obtaining the convalescent sample.

### Specimen collection and processing

Acute COVID-19 pediatric samples were collected between days 0–12 of a positive SARS-CoV-2 test (median 1 day, IQR 1, 3 days). MIS-C patient samples were collected between days 0–17 of hospitalization (median 2 days, IQR 1, 3 days). Convalescent pediatric samples were collected between days 30–110 after their positive PCR test (median 59 days, IQR 50, 77 days). Pediatric samples were collected April 2020 through March 2021 (see Table S2).

Fresh blood was collected into sodium heparin, EDTA, or no additive vacutainers and centrifuged at 1300 x *g* (RCF) for 10 min at room temperature. Plasma or serum was aliquoted and frozen at −80 °C. If a fresh blood sample was not obtained upon enrollment, residual specimens from clinical testing (lithium heparin plasma or serum) were retrieved. Samples were heat-treated at 56 °C for 1 h and refrozen at −80 °C prior to assay.

### Lentivirus pseudovirion neutralization assay

Antibody preparations were evaluated by a qualified SARS-CoV-2 pseudovirus neutralization assay (PsVNA) using WA1 strain and VOCs: Alpha (B.1.1.7), Beta (B.1.351), Gamma (P.1), Delta (B.1.617.2), and Omicron (B.1.1.529) (Supplementary Table S3). The PsVNA using 293-ACE2-TMPRSS2 cell line was described previously^18,21^. SARS-CoV-2 neutralizing activity measured by pseudovirion neutralization assay (PsVNA) correlated with PRNT (plaque reduction neutralization test with authentic SARS-CoV-2 virus) in previous studies^17,18^.

Briefly, human codon-optimized cDNA encoding SARS-CoV-2 S glycoprotein of the WA-1 or the VOCs was synthesized by GenScript and cloned into eukaryotic cell expression vector pcDNA 3.1, between the *BamH*I and *Xho*I sites. Pseudovirions were produced by co-transfection Lenti‐X‐ 293T cells with psPAX2(gag/pol), pTrip-luc lentiviral vector, and pcDNA 3.1 SARS-CoV-2-spike-deltaC19/spike plasmid of VOC, using Lipofectamine 3000. The supernatants were. harvested at 48 h post transfection, filtered through 0.45 µm membranes, and titrated using 293T-ACE2-TMPRSS2 cells (HEK 293T cells that express ACE2 and TMPRSS2 proteins)^34^.

Neutralization assays were performed as previously described^21,35^. For the neutralization assay, 50 µL of SARS-CoV-2 S pseudovirions (counting ~200,000 relative light units) were pre-incubated with an equal volume of medium containing serial dilutions (20−, 60−, 180−, 540−, 1,620−, 4,860−, 14,580−, and 43,740-fold dilution at the final concentration) of heat-inactivated serum at room temperature for 1 h. Then 50 µL of virus-antibody mixtures were added to 293T-ACE2-TMPRSS2 cells (10^4^ cells/50 μL)^34^ in a 96-well plate. The input virus with all SARS-CoV-2 strains used in the current study were the same (2 × 10^5^ relative light units/50 µL/well). After a 3 h incubation, fresh medium was added to the wells. Cells were lysed 24 h later, and luciferase activity was measured using One-Glo luciferase assay system (Promega, Cat# E6130). The assay of each serum was performed in duplicate, and the 50% neutralization titer was calculated using Prism 9 (GraphPad Software).

The PsVNA is a qualified assay where all samples are run with set of internal standards in every plate and conforms with assay performance. Evaluation of serum or plasma (collected with various anticoagulants: sodium heparin, lithium heparin, and EDTA), and other body fluids demonstrated no impact of sample matrix type in the neutralization assay. Samples were fresh and not freeze-thawed. Controls included cells only, viruses without any antibody and positive sera. The limit of detection for the neutralization assay is 1:20. We did not observe any batch effects as variations for replicates within the assays are <7% for PsVN assays.

### Seroreactivity of post-vaccination samples to SARS-CoV-2 nucleocapsid by ELISA

96-well Immulon plates were coated with 50 ng/100 µL of recombinant nucleocapsid from WA1/2020 in PBS overnight at 4oC. Starting at a 1:20 dilution, serum samples were serially diluted 5-fold and applied to the coated well for 1 hr at ambient temperature. Serum samples were assayed in duplicate. After three washes with PBS/0.05% Tween 20, bound human IgG antibodies were detected with 1:5000 dilution of HRP-conjugated anti-human IgG Fc-specific antibody (Jackson Immuno Research). After 1 h, plates were washed PBST followed by PBS, and o-Phenylenediamine dihydrochloride (OPD) was added for 10 min. Absorbance was measured at 492 nm. End-point titer was determined as 2-fold above the average of the absorbance values of the binding of serum samples to blank control wells. The end-point titer is reported as the serum dilution that was above this cutoff and was calculated using Prism 9 (GraphPad Software).

### Statistical analysis

All experimental data were analyzed using R statistical software (version 4.1.2). Absolute measurements were log2-transformed prior to analysis. The statistical analysis tested for significant differences in neutralization titer measurements between (a) different age groups (<5 years old, 5–11 years old, and 12–21 years old) within a fixed patient disease category (acute COVID-19, convalescent COVID-19, and MIS-C), (b) different patient disease categories within a fixed age group, and (c) between variants/mutations within a fixed age and disease category group. Statistical significance was assessed based on permutation testing, avoiding distribution assumptions about the (log) titer measurements^36^. For the tests between age groups and disease categories, log titer measurements were permuted between the samples in the respective groups/categories. For the tests between variants/mutations, log titer measurements were randomly re-assigned to variants/mutations within samples. Two-sided empirical p-values were estimated as the proportion of 1,000,000 random permutations with larger absolute value mean difference of log titer measurements.

### Reporting summary

Further information on research design is available in the Nature Research Reporting Summary linked to this article.

## Supplementary information

Supplementary Information

Peer Review File

Reporting Summary

## Data Availability

All data are shown in the manuscript figures and supplementary information. All the data generated in this study are provided in the Source Data file

## Source data

Source Data
