# Supplementary material for: Cross-reactive immunity against the SARS-CoV-2 Omicron variant is low in pediatric patients with prior COVID-19 or MIS-C
Source: Nat Commun. 2022 May 27;13:2979. doi: 10.1038/s41467-022-30649-1 (PMC9142524; doi:10.1038/s41467-022-30649-1)
Supplement: Supplementary file 1 — Supplementary Information [file 41467_2022_30649_MOESM1_ESM.pdf]

SUPPLEMENTAL INFORMATION (Cross-reactive immunity against the SARS-CoV-2 Omicron variant is low in pediatric patients with prior COVID-19 or MIS-C)

Overcoming COVID-19 Investigators, Sites Listed by State including Additional Non-author Contributors to be listed in PubMed

Alabama: Children's of Alabama, Birmingham. Michele Kong, MD.

Arkansas: Arkansas Children's Hospital, Little Rock. Ronald C. Sanders Jr., MD, MS; Katherine Irby, MD.

California: UCSF Benioff Children's Hospital Oakland, Oakland. Natalie Z. Cvijanovich, MD.

California: UCSF Benioff Children's Hospital, San Francisco. Matt S. Zinter, MD

Colorado: Children's Hospital Colorado, Aurora. Aline B. Maddux, MD, MSCS; Christina M. Osborne, MD; Sara Shankman, DNP, CPNC-AC; Emily Port, BA.

Georgia: Children's Healthcare of Atlanta at Egleston, Atlanta. Keiko M. Tarquinio, MD.

Illinois: Ann & Robert H. Lurie Children's Hospital of Chicago, Chicago. Kelly N. Michelson, MD, MPH; Bria M. Coates, MD.

Indiana: Riley Hospital for Children, Indianapolis. Courtney M. Rowan, MD, MS.

Massachusetts: Boston Children's Hospital, Boston. Adrienne G. Randolph, MD; Tanya Novak, PhD, Sabrina R. Chen; Suden Kucukak, MD; Margaret M. Newhams, MPH; Jeni Melo, BS; Maya Levine-Ritterman, MA; Hye Kyung Moon, MA; Megan Elkins, MSc; Sabrina Weeks, MMSc; Alan Nguyen, MD, Saddiq Habiballah, MD, Amer Al-Musa, MD.

Michigan: University of Michigan CS Mott Children's Hospital, Ann Arbor. Heidi R. Flori, MD, FAAP, Mary K. Dahmer, PhD

Minnesota: University of Minnesota Masonic Children's Hospital, Minneapolis, Janet R. Hume, MD, PhD.

Mississippi: University of Mississippi Medical Center, Jackson: Charlotte V. Hobbs, MD; Lora Martin, NP; Lacy Malloch, BSc; John M. Williams, MSc; Gurbaksh Singh, MSc; Urita Agana, BSc; Kayla Patterson, MSc.

Missouri: Children's Mercy Hospital, Kansas City. Jennifer E. Schuster, MD.

New Jersey: Cooperman Barnabas Medical Center, Livingston. Shira J. Gertz, MD.

North Carolina: University of North Carolina at Chapel Hill, Chapel Hill. Stephanie P. Schwartz, MD; Tracie C. Walker, MD.

Ohio: University Hospitals Rainbow Babies and Children's Hospital, Cleveland. Steven L. Shein, MD; Amanda N. Lansell, MD.

Ohio: Cincinnati Children's Hospital, Cincinnati. Mary A. Staat, MD, MPH.

Pennsylvania: Children's Hospital of Philadelphia, Philadelphia. Julie C. Fitzgerald, MD, PhD, MSCE; Jenny L. Bush RN, BSN; Ryan H. Burnett, BS.

South Carolina: MUSC Children's Health, Charleston. Elizabeth H. Mack, MD, MS.; Laura Smallcomb, MD.

Tennessee: Monroe Carell Jr. Children's Hospital at Vanderbilt, Nashville. Natasha B. Halasa, MD, MPH.

Texas: Texas Children's Hospital, Houston. Laura L. Loftis, MD.

Utah: Primary Children's Hospital, Salt Lake City. Hillary Crandall, MD, PhD; Evan Heller, CCRC.

Taking on COVID-19 Together Investigators Non-author Contributors to be listed in PubMed

These individuals provided study oversight, identified and enrolled patients, collected samples, processed samples, and otherwise contributed to the acute, MIS-C and convalescent sample and data collection at Boston Children's Hospital with oversight from the Institutional Center for Clinical and Translational Research (ICCTR).

David A. Williams, MD, Lucinda Williams, DNP, RN, PNP, Myriam Armant, PhD, Benjamin Raby, MD, Maggie Malsch, MSN, RN, Leah Cheng, MA, Allyson McCrary, BA, Sarah Steltz, MPH Michael Lorrey-Parena, RN, Debra Morley, PhD, Catherine Dempsey, RN, BSN, Christopher Wan, BA, Kelly Zakar, RN, MSN, Ivy Dang, RN, BSN, Ashley Kuniholm, CIP, Erica Denhoff, Felicia Ciuculescu, MD, Alejandra Rincon-Bolivar, BA.

Supplementary Table S1: Patient demographics and clinical data

**Patient Demographics and Characteristics** N (%)	**Acute**			**MIS-C**			**Convalescent**		
	**< 5 Years (n=22)**	**5-11 Years (n=14)**	**12-21 Years (n=26)**	**< 5 Years (n=22)**	**5–11 Years (n=20)**	**12-21 Years (n=23)**	**< 5 Years (n=11)**	**5-11 Years (n=17)**	**12-21 Years (n=22)**
**Male**	11 (50)	8 (57)	11 (42)	10 (45)	12 (60)	15 (65)	3 (27)	12 (71)	11 (50)
**Age,** Median (IQR)	0.4 (0.1, 1.8)	8.2 (7.1, 8.9)	15.9 (14.5, 17.6)	3 (1.5, 4.1)	8.2 (7.2, 10)	14.8 (14, 17)	1.7 (1.2, 2.7)	8.6 (6.7, 9.7)	15.5 (14.7, 19)
**Hispanic or Latino**	8 (36)	5 (36)	11 (42)	7 (32)	7 (35)	3 (13)	4 (36)	5 (29)	10 (45)
**Race**									
White	11 (50)	6 (43)	7 (27)	9 (41)	6 (30)	12 (52)	3 (27)	1 (6)	7 (32)
Black/African American	6 (27)	3 (21)	5 (19)	7 (32)	5 (25)	6 (26)	0	3 (18)	3 (14)
Asian	0	1 (7)	1 (4)	0	0	0	0	0	2 (9)
American Indian or Alaska Native	0	0	0	0	0	0	1 (9)	2 (12)	0
Mixed, Other, Refused or Don't know	5 (23)	4 (29)	13 (50)	6 (27)	9 (45)	5 (22)	7 (64)	11 (65)	10 (45)
**SARS-CoV-2 PCR+**	22 (100)	14 (100)	26 (100)	7 (32)	12 (60)	12 (52)	11 (100)	17 (100)	22 (100)
**Previously Healthy**	16 (73)	3 (21)	6 (23)	19 (86)	11 (55)	12 (52)	6 (55)	4 (24)	9 (41)
**Underlying Conditions***									
Obesity	0	2 (14)	10 (38)	1 (5)	3 (15)	5 (22)	1 (9)	4 (24)	1 (5)
Respiratory disorders	2 (9)	3 (21)	8 (31)	1 (5)	4 (20)	2 (9)	1 (9)	3 (18)	4 (18)
Cardiovascular disorders	2 (9)	1 (7)	3 (12)	0	1 (5)	1 (4)	0	0	1 (5)
Neurologic or neuromuscular	4 (18)	5 (36)	5 (19)	2 (9)	1 (5)	2 (9)	2 (18)	4 (24)	5 (23)
Hematologic disorder	4 (18)	0	3 (12)	0	0	1 (4)	0	3 (18)	0
Gastrointestinal or hepatic	3 (14)	3 (21)	6 (23)	0	0	2 (9)	1 (9)	2 (12)	1 (5)
Metabolic or genetic	3 (14)	2 (14)	13 (50)	1 (5)	4 (20)	5 (22)	1 (9)	6 (35)	4 (18)
Other disorders**	0	4 (29)	9 (35)	0	1 (5)	3 (13)	1 (9)	1 (6)	3 (14)
**ICU Admission**	8 (36)	6 (43)	14 (54)	16 (73)	14 (70)	17 (74)			
**Received any respiratory support**	6 (27)	5 (36)	16 (62)	10 (45)	10 (50)	12 (52)			
Mechanical ventilation (invasive/ noninvasive)	3 (14)	3 (21)	6 (23)	2 (9)	1 (5)	5 (22)			
**Median days in-hospital (IQR)**	2.5 (1, 5.8)	3 (2, 5.8)	5.5 (2.3, 7.8)	5 (3.3, 8)	6 (4.8, 9)	6 (5, 9)			
**Hospital mortality**	0	0	0	0	0	0			

* 2 patients deemed 'not previously healthy' but no underlying conditions were disclosed.
** Other underlying conditions include active or prior oncologic issues, autoimmune disorder, renal or urologic dysfunction, and endocrine disorders.

Supplementary Table S2. Timing of pediatric sample collection relative to hospital admission or PCR positive test.

Stats	Acute Inpatient	PCR + Date	MIS-C	Admit Date	Estimated Exposure Date*	Convalescent Outpatient	PCR Positive Date	Sample Collection Date
**Median**	<5 y	**Nov 2020**	<5 y	**Nov 2020**	**Oct 2020**	<5 y	**Oct 2020**	**Dec 2020**
IQR (25%)	n=22	Oct 2020	n=22	Aug 2020	July 2020	n=11	May 2020	Aug 2020
IQR (75%)		Dec 2020		Jan 2021	Dec 2020		Dec 2020	Feb 2021
Range, First		June 2020		Apr 2020	Mar 2020		May 2020	July 2020
Range, Last		Jan 2021		Feb 2021	Jan 2021		Jan 2021	Mar 2021
**Median**	5-11 y	**Oct 2020**	5-11 y	**Oct 2020**	**Sept 2020**	5-11 y	**Oct 2020**	**Nov 2020**
IQR (25%)	n=14	Aug 2020	n=20	June 2020	May 2020	n=17	May 2020	Aug 2020
IQR (75%)		Dec 2020		Jan 2021	Dec 2020		Nov 2020	Jan 2021
Range, First		May 2020		Apr 2020	Mar 2020		Apr 2020	June 2020
Range, Last		Jan 2021		Feb 2021	Jan 2021		Dec 2020	Feb 2021
**Median**	12-21 y	**Sept 2020**	12-21 y	**Dec 2020**	**Nov 2020**	12-21 y	**Oct 2020**	**Dec 2020**
IQR (25%)	n=26	July 2020	n=23	Oct 2020	Sept 2020	n=22	**Oct 2020**	**Nov 2020**
IQR (75%)		Nov 2020		Jan 2021	Dec 2020		Nov 2020	Jan 2021
Range, First		Apr 2020		May 2020	Apr 2020		Apr 2020	Aug 2020
Range, Last		Jan 2021		Feb 2021	Jan 2021		Jan 2021	Mar 2021

*30 days prior to hospital admission

Supplementary Table S3: SARS-CoV-2 variants mutations introduced in the spike plasmid for production of SARS-CoV-2 pseudovirions for analysis in PsVNA.

SARS-CoV-2 variant	Mutations constructed in the spike plasmids
**Alpha (B.1.1.7)**	H69-V70del, Y144del, N501Y, A570D, D614G, P681H, T716I, S982A, and D1118H
**Beta (B.1.351)**	L18F, D80A, D215G, L242-244del, R246I, K417N, E484K, N501Y, D614G, and A701V
**Gamma (P.1)**	L18F, T20N, P26S, D138Y, R190S, K417T, E484K, N501Y, H655Y, T1027I, D614G, V1176F
**Delta (B.1.617.2)**	T19R,G142D,E156del,F157del,R158G,L452R,T478K,D614G,P681R,D950N
**Omicron (B.1.1.529)**	A67V, H69-70del, T95I, G142D, V143-145del, Y145D, N211del, L212I, ins214EPE, G339D, S371L, S373P, S375F, K417N, N440K, G446S, S477N, T478K, E484A, Q493R, G496S, Q498R, N501Y, Y505H, T547K, D614G, H655Y, N679K, P681H, N764K, D796Y, N856K, Q954H, N969K, L981F

Supplementary Figure S1

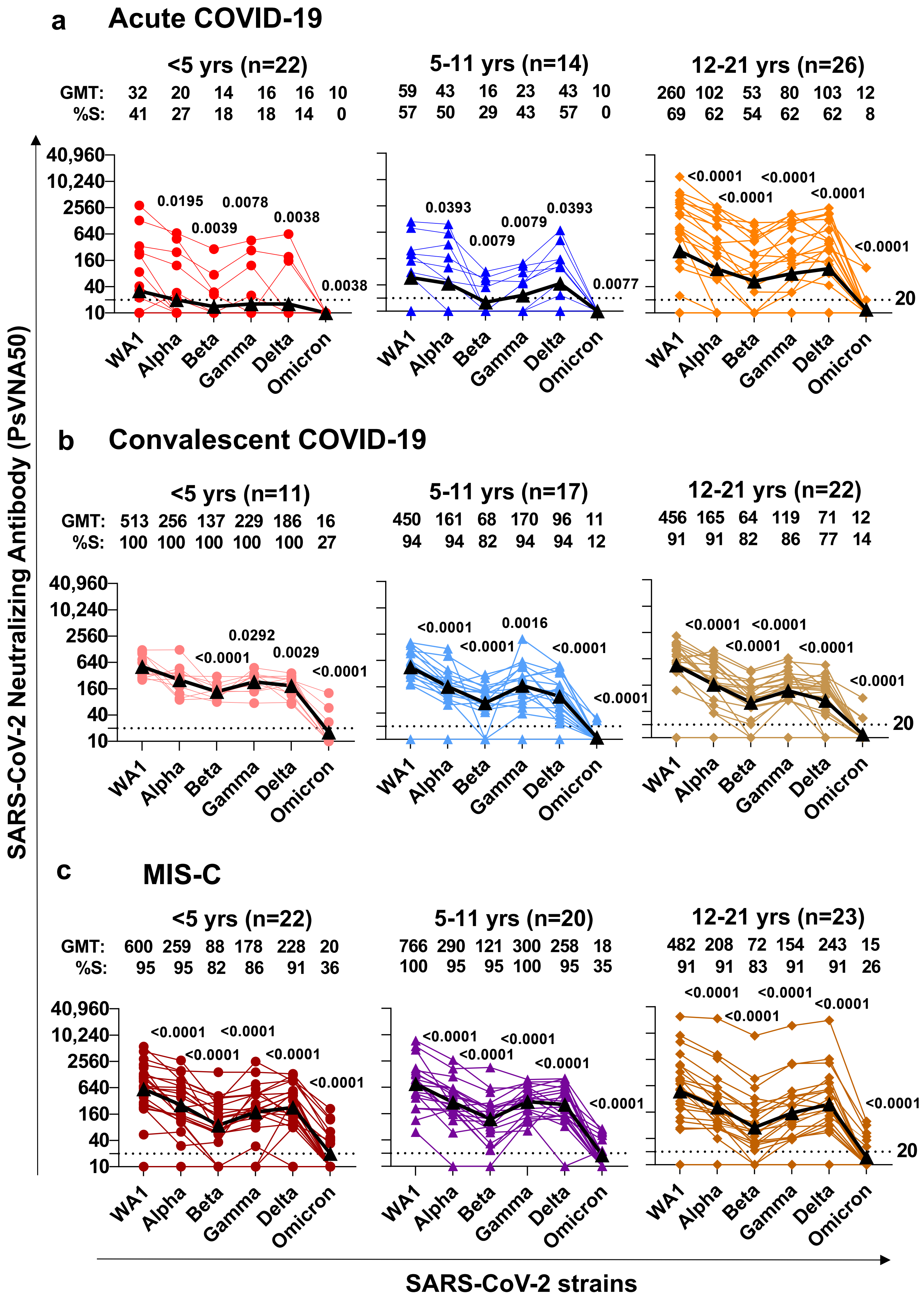

Supplementary Figure S1. Neutralizing antibody titers of serum/plasma from children with COVID-19 or MIS-C against SARS-CoV-2 WA1 and VOCs.

(A-C) SARS-CoV-2 neutralizing antibody titers in 177 children sera/plasma with either acute COVID-19 (a), convalescent COVID-19 (b) or MIS-C (c) as determined by pseudovirus neutralization assay in 293-ACE2-TMPRSS2 cells with SARS-CoV-2 WA1 and VOCs: Alpha (B.1.1.7), Beta (B.1.351), Gamma (P.1), Delta (B.1.617.2) and Omicron (B.1.1.529). PsVNA50 (50% neutralization titer) titers for younger children (<5 years), 5-11 years and adolescents (12-21 years). PsVNA50 GMTs are shown as black triangles and are presented for each age group against the SARS-CoV-2 WA1 and VOCs on top of the panel. Sample with PsVNA50 titer $\geq$ 1:20 is defined as seropositive. Percent seropositivity (%S) for each group was calculated as number of seropositive samples in the group divided by total number of samples x 100 in the group. The PsVNA is a qualified assay where all samples are run with set of internal standards in every plate of the neutralization assay and conforms with assay performance. All PsVNA experiments were performed in duplicate and the researchers performing the assay were blinded to sample identity. The variations for duplicate runs were <7%. The data shown are average values of two experimental runs. Statistical differences were analyzed in R (version 4.1.2) using a permutation-based approach and the two-sided statistically significant p-values are shown. The p-values are not corrected for multiple comparisons.

Supplementary Figure S2

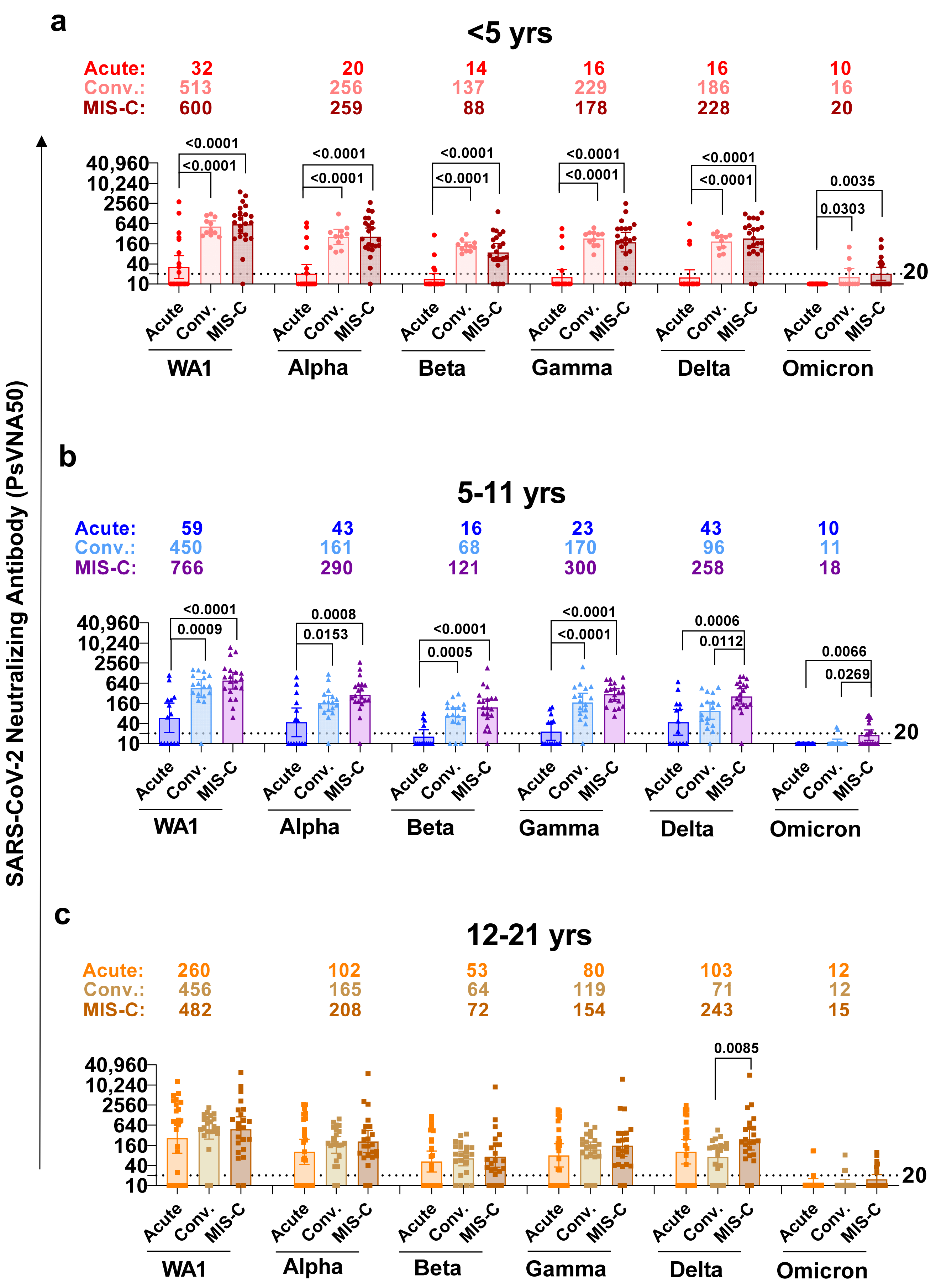

Supplementary Figure S2: Comparison of neutralizing antibodies in different age group children with acute COVID-19 vs convalescent COVID-19 vs MIS-C against various SARS-CoV-2 strains.

Geometric mean titer (GMT) values $\pm$ 95% CI of PsVNA50 (50% neutralization) titers for sample from young children (<5 years; in a), school-age children (5-11 years, in b), and adolescent (12-21 years, in c), with either acute COVID-19 (in red), convalescent COVID-19 (in black) or MIS-C (in blue), against SARS-CoV-2 WA1 or the VOCs Alpha (B.1.1.7), Beta (B.1.351), Gamma (P.1), Delta (B.1.617.2) and Omicron (B.1.1.529) by pseudovirus neutralization assay in 293-ACE2-TMPRSS2 cells. The GMT values for PsVNA50 titers against the SARS-CoV-2 WA1 and VOCs and are color coded for each of the disease group matching the colors in the graph. Data shown for acute COVID-19 patients, either children with acute COVID-19; <5 years;(n=22), 5-11 years (n=14), 12-21 years (n=26); or convalescent COVID-19 patients, either children with convalescent COVID-19; <5 years (n=11), 5-11 years (n=17), 12-21 years (n=22), or children with MIS-C; <5 years (n=22), 5-11 years (n=20) and 12-21 years (n=23). All PsVNA experiments were performed twice and the researchers performing the assay were blinded to sample identity. The variations for duplicate runs was <7%. The data shown are average values of two experimental runs. Statistical differences were analyzed in R (version 4.1.2) using a permutation-based approach and the two-sided statistically significant p-values are shown. The p-values are not corrected for multiple comparisons.
