# Supplementary material for: Cross-reactive immunity against the SARS-CoV-2 Omicron variant is low in pediatric patients with prior COVID-19 or MIS-C
Source: Nat Commun. 2022 May 27;13:2979. doi: 10.1038/s41467-022-30649-1 (PMC9142524; doi:10.1038/s41467-022-30649-1)
Supplement: Supplementary file 3 — Reporting Summary [file 41467_2022_30649_MOESM3_ESM.pdf]

nature research

Corresponding author(s): Surender Khurana

Last updated by author(s): Apr 25, 2022

Reporting Summary

Nature Research wishes to improve the reproducibility of the work that we publish. This form provides structure for consistency and transparency in reporting. For further information on Nature Research policies, see our Editorial Policies and the Editorial Policy Checklist.

Statistics

For all statistical analyses, confirm that the following items are present in the figure legend, table legend, main text, or Methods section.

n/a	Confirmed	
☐	☒	The exact sample size (*n*) for each experimental group/condition, given as a discrete number and unit of measurement
☐	☒	A statement on whether measurements were taken from distinct samples or whether the same sample was measured repeatedly
☐	☒	The statistical test(s) used AND whether they are one- or two-sided<br>*Only common tests should be described solely by name; describe more complex techniques in the Methods section.*
☐	☒	A description of all covariates tested
☐	☒	A description of any assumptions or corrections, such as tests of normality and adjustment for multiple comparisons
☐	☒	A full description of the statistical parameters including central tendency (e.g. means) or other basic estimates (e.g. regression coefficient) AND variation (e.g. standard deviation) or associated estimates of uncertainty (e.g. confidence intervals)
☐	☒	For null hypothesis testing, the test statistic (e.g. *F*, *t*, *r*) with confidence intervals, effect sizes, degrees of freedom and *P* value noted<br>*Give P values as exact values whenever suitable.*
☐	☒	For Bayesian analysis, information on the choice of priors and Markov chain Monte Carlo settings
☐	☒	For hierarchical and complex designs, identification of the appropriate level for tests and full reporting of outcomes
☐	☒	Estimates of effect sizes (e.g. Cohen's *d*, Pearson's *r*), indicating how they were calculated

Our web collection on statistics for biologists contains articles on many of the points above.

Software and code

Policy information about availability of computer code

Data collection	Antibody data was collected in MS Excel version 16.57.
Data analysis	Antibody titers were calculated using Prism 9.3.1 (GraphPad Software). Statistical analysis were performed using R statistical software (version 4.1.2), and all information is provided the statistical methods section.

For manuscripts utilizing custom algorithms or software that are central to the research but not yet described in published literature, software must be made available to editors and reviewers. We strongly encourage code deposition in a community repository (e.g. GitHub). See the Nature Research guidelines for submitting code & software for further information.

Data

Policy information about availability of data

All manuscripts must include a data availability statement. This statement should provide the following information, where applicable:

- Accession codes, unique identifiers, or web links for publicly available datasets
- A list of figures that have associated raw data
- A description of any restrictions on data availability

All data are shown in the manuscript figures and supplementary information. The complete dataset for this study are provided in the Source Data file.

Field-specific reporting

Life sciences study design

All studies must disclose on these points even when the disclosure is negative.

Sample size	All available samples were analyzed in this study
Data exclusions	No data was excluded
Replication	Neutralization and ELISA were performed twice by independent researchers in the lab. The replications were successful. The variation in duplicate experimental runs was <7% for neutralization and <8% for ELISA.
Randomization	All samples from the children and adult patients were analyzed in this study. The study was non-randomized performed during the pandemic on outpatient and inpatients. Initially, no patient information was provided, and all the immune analyses were conducted blindly by the researcher's performing the assays. The participants were assigned in each experimental group based on their hospitalization status and clinical outcome.
Blinding	Experiments were performed by different investigators, who were blinded to sample identity.

Reporting for specific materials, systems and methods

We require information from authors about some types of materials, experimental systems and methods used in many studies. Here, indicate whether each material, system or method listed is relevant to your study. If you are not sure if a list item applies to your research, read the appropriate section before selecting a response.

Materials & experimental systems

n/a	Involved in the study	
☐	☒	Antibodies
☐	☒	Eukaryotic cell lines
☒	☐	Palaeontology and archaeology
☒	☐	Animals and other organisms
☐	☒	Human research participants
☒	☐	Clinical data
☒	☐	Dual use research of concern

Methods

n/a	Involved in the study	
☒	☐	ChIP-seq
☒	☐	Flow cytometry
☒	☐	MRI-based neuroimaging

Antibodies

Antibodies used	HRP-conjugated goat anti-human IgG-Fc specific antibody (Cat no #709-005-098) were purchased from Jackson Immuno Research.
Validation	The secondary antibodies were characterized by the manufacturer (https://www.jacksonimmuno.com/)

Eukaryotic cell lines

Policy information about cell lines

Cell line source(s)	Lenti-X- 293T cells were obtained from Takara Bio (Cat. No. 632180). 293_ACE2_TMPRSS2 cells were generated and sourced from the lab of Carol Weiss at FDA (Neerukonda, S.N. et al. (2021) PLoS One 16, e0248348).
Authentication	Cell lines were checked for expression of ACE2 and validated by FACS analysis. None of the cell lines were authenticated by karyotyping or other genomic techniques. Reference: Neerukonda, S.N. et al. Establishment of a well-characterized SARS-CoV-2 lentiviral pseudovirus neutralization assay using 293T cells with stable expression of ACE2 and TMPRSS2. PLoS One 16, e0248348 (2021).
Mycoplasma contamination	Negative for Mycoplasma
Commonly misidentified lines (See ICLAC register)	No misidentified cell lines were used in the study.

Human research participants

Policy information about studies involving human research participants

Population characteristics	Participants in this study were aged 0-21 years old. Individuals of any gender that were exposed to SARS-CoV-2 or COVID-19 vaccination were eligible for the study.
Recruitment	All patients SARS-CoV-2 infection or naive SARS-CoV-2 vaccinees were eligible without any specific selection criteria. Samples were collected from children following informed consent and assent from at least one parent or legal guardian as appropriate for age to participate in the study during the pandemic. Pediatric participants were recruited in a Overcoming COVID-19 multicenter network study or the UCSD study without any selection bias.
Ethics oversight	Children and adolescents, 0 to 21 years of age, with confirmed SARS-CoV-2 positive PCR or antibody testing were enrolled into an IRB-approved specimen collection protocol following informed consent and assent as appropriate for age. The Overcoming COVID-19 Network studies severe complications of COVID-19 in children and adolescents consists of a subgroup of 20 sites in 18 U.S. states. Study sites relied on a single IRB at Boston Children's Hospital under Protocol Number #IRB-P00033157, and informed consent was obtained from at least one parent or legal guardian. Samples from the Boston Children's Hospital COVID-19 Biobank included consented patient samples and deidentified samples obtained with IRB approved waiver of consent.<br>Vaccinated healthy control samples from University of California San Diego (UCSD) were obtained following parental and subject consent and assent as appropriate. The protocol was approved by the IRB at UCSD (#140220).<br>Samples were tested in different antibody assays with approval from the U.S. Food and Drug Administration's Research Involving Human Subjects Committee (FDA-RIHSC) under exemption protocol '252-Determination-CBER-2020-04-02'.

Note that full information on the approval of the study protocol must also be provided in the manuscript.
