# Supplementary material for: Cross-reactive immunity against the SARS-CoV-2 Omicron variant is low in pediatric patients with prior COVID-19 or MIS-C
Source: Nat Commun. 2022 May 27;13:2979. doi: 10.1038/s41467-022-30649-1 (PMC9142524; doi:10.1038/s41467-022-30649-1)
Supplement: Supplementary file 2 — Peer Review File [file 41467_2022_30649_MOESM2_ESM.pdf]

nature portfolio

Peer Review File

Cross-reactive immunity against the SARS-CoV-2 Omicron variant is low in pediatric patients with prior COVID-19 or MIS-C

Open Access This file is licensed under a Creative Commons Attribution 4.0 International License, which permits use, sharing, adaptation, distribution and reproduction in any medium or format, as long as you give appropriate credit to the original author(s) and the source, provide a link to the Creative Commons license, and indicate if changes were made. In the cases where the authors are anonymous, such as is the case for the reports of anonymous peer reviewers, author attribution should be to 'Anonymous Referee' followed by a clear attribution to the source work. The images or other third party material in this file are included in the article's Creative Commons license, unless indicated otherwise in a credit line to the material. If material is not included in the article's Creative Commons license and your intended use is not permitted by statutory regulation or exceeds the permitted use, you will need to obtain permission directly from the copyright holder. To view a copy of this license, visit http://creativecommons.org/licenses/by/4.0/.

Editorial Note: This manuscript has been previously reviewed at another journal that is not operating a transparent peer review scheme. This document only contains reviewer comments and rebuttal letters for versions considered at Nature Communications.

Reviewers' Comments:

Reviewer #2:
Remarks to the Author:
The authors have addressed most comments from both reviewers. The inclusion of vaccinated older children as controls for the younger unvaccinated children, however, remains a limitation.

Reviewers' Comments:

Reviewer #2:

The authors have addressed most comments from both reviewers. The inclusion of vaccinated older children as controls for the younger unvaccinated children, however, remains a limitation.

Response: We thank the reviewer for the positive response. The limitation of vaccinated children is acknowledged in the discussion section:

Lines 311-314: Another limitation is cohort of vaccinated children is small, as well as skewed towards the older (>15) age group. However, the neutralizing antibody response measured in our study mimics to those observed in younger children against the vaccine-matched strain28.
